# Neutrophil extracellular traps induce thrombogenicity in severe carotid stenosis

**DOI:** 10.1002/iid3.466

**Published:** 2021-06-08

**Authors:** Shihua Zhang, Mengfan Guo, Qianzi Liu, Jingfeng Liu, Yankun Cui

**Affiliations:** ^1^ Department of Neurosurgery of the First Affiliated Hospital Jiamusi University Jiamusi China; ^2^ Department of Pathology of the First Affiliated Hospital Jiamusi University Jiamusi China; ^3^ Department of Pharmacy Jiamusi University Jiamusi China; ^4^ Department of Outpatient of the First Affiliated Hospital Jiamusi University Jiamusi China

**Keywords:** endothelial cells, severe carotid stenosis, neutrophil extracellular traps, thrombosis, tissue factor

## Abstract

**Background:**

Severe carotid stenosis is a common cause of stroke. In addition, previous clinical studies revealed that patients symptomatic of carotid stenosis suffer from increased episodes of stroke compared with their asymptomatic counterparts. However, the mechanism underlying these differences in the recurrence of stroke remains unclear.

**Objective:**

The present study aimed to evaluate the levels of neutrophil extracellular traps (NETs) in the plasma of patients with severe carotid stenosis and investigate whether NETs induced procoagulant activity (PCA) in severe carotid stenosis. The study also sought to investigate the interactions between platelets or endothelial cells (ECs) and NETs.

**Methods:**

The levels of NETs in plasma were quantified using enzyme‐linked immunosorbent assay (ELISA). In addition, NETting neutrophils and neutrophil‐platelet aggregates were detected through flow cytometry. On the other hand, the morphology of NETs formation and endothelial cells were analyzed through confocal microscopy. Finally, the procoagulant activity (PCA) of NETs and endothelial cells were assessed through ELISA and fibrin formation.

**Results:**

Patients with symptomatic carotid stenosis patients had significantly higher levels of NETs markers compared with their asymptomatic counterparts and healthy subjects. In addition, increased levels of neutrophil‐platelet aggregates induced the generation of NETs in patients with symptomatic carotid stenosis. Moreover, NETs contributed to PCA through tissue factor (TF), in patients with carotid stenosis. Furthermore, NETs disrupted the endothelial barrier and converted endothelial cells (ECs) into PCA to enhance the PCA in patients with carotid stenosis.

**Conclusions:**

The current study revealed differences in the levels of NETs in the plasma of symptomatic and asymptomatic patients suffering from carotid stenosis. The study also uncovered the interaction between NETs and thrombogenicity in carotid stenosis. Therefore, inhibiting NETs may be a potential biomarker and therapeutic target for recurring stroke in severe carotid stenosis.

## Graphical abstract

The plasma levels of neutrophil extracellular traps (NETs) are increased in samples from severe carotid stenosis patients. • Platelets (PLT) initiate the formation of NETs in severe carotid stenosis patients. • NETs induce hypercoagulation state in severe carotid stenosis patients and convert endothelial cells (ECs) to procoagulant phenotype.

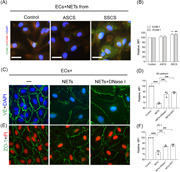

## 1 INTRODUCTION

Stroke is a significant challenge to public health worldwide with an estimated 16 million new cases every year,^1^ making it the second leading cause of death and third leading cause of disability in adults across the globe.^2^ In addition, the extracranial internal carotid artery atherosclerotic occlusive disease is a common cause of stroke, accounting for about 7%–18% of all the stroke incidences.^3^, ^4^, ^5^ Moreover, previous studies suggested that asymptomatic carotid stenosis within the range of 60%–99% translates the annual risk of ipsilateral ischemic stroke to between 0.34% and 2%.^6^, ^7^, ^8^, ^9^, ^10^ This is much lower than the recently observed risk of ipsilateral stroke recurrence in severe symptomatic patients. However, the mechanism underlying this disparity in the incidence of stroke recurrence is yet to be fully elucidated.

It is well known that atherosclerosis is an inflammatory disease and monocyte‐derived macrophages were recognized to be the most abundant cell type during the progression of atherosclerosis.^11^ However, increasing evidence suggests that neutrophils are not only classic antimicrobial immune cells but also major players in the development of atherosclerosis.^12^, ^13^, ^14^, ^15^ According to previous studies, neutrophils existed in carotid plaques mainly in the form of neutrophil extracellular traps (NETs).^16^, ^17^, ^18^, ^19^ Notably, NETs are web‐like structures released by activated neutrophils and are composed of DNA, histones, and neutrophil granule proteins such as elastase, myeloperoxidase, and cathepsin G.^20^, ^21^, ^22^ Additionally, NETs were reported to be an important constituent of stroke thrombi and contributed to thrombogenicity in stroke patients.^23^, ^24^, ^25^, ^26^ Moreover, severe carotid stenosis was reported to be a leading cause of stroke recurrence.^10^, ^27^ However, little is known about the presence and the potential role of NETs in severe carotid stenosis. According to previous studies, NETs could induce the apoptosis of endothelial cells (ECs) and drive endothelial to‐mesenchymal transition.^28^, ^29^, ^30^, ^31^ However, the potential interaction between NETs and ECs in severe carotid stenosis has not been studied.

In this study, the results showed that the plasma levels of the markers of NETs were higher in patients with symptomatic carotid stenosis compared with their asymptomatic counterparts and healthy controls. The study also evaluated the potential interactions between activated platelets (PLTs) and NETs and investigated the contribution of NETs to procoagulant activity (PCA) in carotid stenosis. Furthermore, the study showed that NETs could induce endothelial barrier dysfunction to exacerbate thrombogenicity. These novel findings therefore uncovered the crucial role of NETs in the PCA of severe carotid stenosis, providing a promising therapeutic strategy for the prevention of vascular complications from severe carotid stenosis.

## 2 MATERIALS AND METHODS

### 2.1 Patients

The study selected 12 healthy individuals, 13 patients with asymptomatic carotid stenosis, and 12 with symptomatic carotid stenosis. The main clinical characteristics of the patients and healthy controls are shown in Table 1. The patients were admitted to the First Affiliated Hospital of Jiamusi University between October 2018 and November 2020. Asymptomatic carotid stenosis was defined as: patients with moderate (50%–69%) or severe (70%–99%) carotid stenosis and never had transient ischemic attacks (TIA) or symptoms of stroke or had no history of TIA or stroke in the past 3 years. On the other hand, symptomatic patients were considered to be those who had experienced a TIA or ischemic stroke in the vascular territory supplied by a moderate (50%–69%) or severe (70%–99%) ipsilateral carotid artery stenosis within the past 4 weeks.^32^, ^33^ *The exclusion criteria were patients had liver or renal failure, autoimmune disease hematological disease, infections, malignancy or was pregnant*.

**Table 1:** The main clinical and laboratory features of 12 healthy subjects and 25 patients diagnosed with severe carotid stenosis

Characteristics	Control (*n* = 12)	ASCS (*n* = 13)	SSCS (*n* = 12)
Age (years)	35.07 ± 0.5	35.57 ± 4.0	43.63 ± 0.5
Male (*n*, %)	50%	30.25%	66.7%
WBC (×10^9^)	3.78 ± 1.12	3.85 ± 2.08	3.92 ± 1.73
Neutrophils(%)	65.62 ± 9.43	65.34 ± 7.32	71.45 ± 5.26*
Monocytes (%)	2.39 ± 1.12	2.40 ± 1.06	2.38 ± 1.05
Lymphocytes(%)	15.34 ± 7.55	15.42 ± 7.22	15.52 ± 6.78
Esoinophils(%)	0.30 ± 0.45	0.29 ± 0.35	0.30 ± 0.42
Basophils(%)	0	0	0
Erythrocytes(×10^12^/L)	3.87 ± 0.77	3.84 ± 0.67	3.88 ± 0.49
Hg (g/L)	122 ± 15.4	121 ± 13.6	122 ± 12.5
PLT (×10^9^)	244 ± 42.5	245 ± 38.5	242 ± 29.5
PT (s)	12.2 ± 1.0	12.1 ± 0.9	12.2 ± 0.9
APTT (s)	33.3 ± 2.1	32.7 ± 1.9	33.0 ± 1.5
d‐dimer (mg/L)	348.56 ± 4.1	345.38 ± 5.0	352.35 ± 5.6
Fibrinogen (mg/L)	2.66 ± 0.58	2.67 ± 0.65	2.71 ± 0.47

*Note*: Data are presented as numbers (percentages) or the median ± *SD*. The main clinical and laboratory features of 12 healthy subjects and 25 patients diagnosed with severe carotid stenosis.

Abbreviations: ASCS, asymptomatic sever carotid stenosis: Hb, hemoglobin; PLTs, platelets; SSCS, symptomatic sever carotid stenosis; WBC, white blood cells.

* *p* < .05 versus healthy control.

This study was approved by the research ethics committee of the First Affiliated Hospital of Jiamusi University and informed consent was obtained from all participants.

### 2.2 Human samples

Plasma was obtained from healthy subjects and patients clinically diagnosed with severe carotid stenosis. The blood from healthy subjects and patients was obtained with informed consent. Thereafter, the human neutrophils separation medium (TBD) was used to separate neutrophils, according to the manufacturer's protocol. On the other hand, platelets were isolated as previously described.^26^ In addition, platelet‐rich plasma (PRP) was prepared by centrifugation at 150*g* for 15 min at room temperature and used immediately after isolation.

### 2.3 Stimulation, quantification and isolation of NETs

To stimulate the NETs, isolated neutrophils were stimulated with 500 nmol/L of Phorbol 12‐myristate 13‐acetate (PMA) for 4 h and NET isolation was performed as follows: after stimulation 4 h, we aspirate and discard the media and wash the bottom of each dish by PBS, collecting solution obtained from washing each dish and centrifuge for 10 min at 450g at 4°C. We divide supernatant into 1.5 ml micro‐centrifuge tubes and spin for 10 min at 18,000g at 4°C. Discard supernatant and resuspend all pellets obtained together in ice cold PBS.^34^ For isolation of NETs from healthy subjects and patients was performed as previously described.^31^ Additionally, quantification of cell‐free DNA (Cf‐DNA) was performed using the Quant‐iT PicoGreen dsDNA Assay Kit (Invitrogen) according to the manufacturer's instructions. To detect the NETs complex, the study used the MPO ELISA kit (Elabscience), the NE ELISA kit (Elabscience) and the citrullinated histone H3 (citH3) ELISA kit (jianglaibio) along with the Quant‐iT PicoGreen dsDNA Assay Kit (Invitrogen), respectively, as previously described.^26^, ^35^

### 2.4 EC stimulation assays

Human umbilical vein endothelial cells (HUVECs) were incubated for 2 h with different concentrations of NETs after which the treated ECs were detected through fibrin formation as previously described.^29^ In the inhibition assays, isolated NETs (0.5 μg/ml) were added to the HUVECs in the presence of DNase I (activated protein C [APC], 100 nM) then incubated for 2 h.

### 2.5 Flow cytometry

To detect the phosphatidylserine (PS) exposure and CD62P(P‐selectin) of platelets, platelets isolated from healthy subjects and patients^26^ were stained with fluorescein isothiocyanate (FITC)‐conjugated bovine lactadherin (Haematologic Technologies, Essex Junction, VT) and anti‐CD62P antibody (Proteintech). Thereafter, isolated neutrophils from patients and healthy donors were incubated with FITC‐conjugated‐CD41 (Bioledgend) and the PE‐conjugated‐CD66b (Bioledgend) antibody before analysis using a flow cytometer, to investigate the presence of neutrophil‐PLTs aggregates. Moreover, isolated neutrophils were fixed and stained with anti‐Histone H3 citrulline (R2 + R8 + R17, ab5103) and anti‐MPO (Proteintech), followed by a goat antirabbit secondary antibody conjugated to Alexa Fluor 488 (Proteintech) and a goat antimouse secondary antibody conjugated to Alexa Fluor 647 (ab150115), before analysis using a flow cytometer, to detect the NETting neutrophils.^36^

### 2.6 Immunofluorescence imaging

To stain the NETs, neutrophils were fixed and stained with DAPI (4',6‐diamidino‐2‐phenylindole), anti‐Histone H3 citrulline (R2 + R8 + R17, ab5103), and anti‐MPO (Proteintech) followed by a goat antirabbit secondary antibody conjugated to Alexa Fluor 594 (Proteintech) and a goat antimouse secondary antibody conjugated to Alexa Fluor 488 (Proteintech). Thereafter, treated NETs were stained with anti‐Tissue factor antibody (Bioledgend) and anti‐MPO (Proteintech) followed by a goat antirabbit secondary antibody conjugated to Alexa Fluor 594 (Proteintech) and a goat antimouse secondary antibody conjugated to Alexa Fluor 488 (Proteintech), to visualize the coagulation factors binding to the NETs. Additionally, ECs cultured on fibronectin‐coated slide flasks were stained with anti‐VE‐cadherin antibody (ab33168) and DAPI or anti‐ZO‐1 antibody (Proteintech) and PI (propidium iodide). All the immunofluorescence images were analyzed through confocal microscopy.

### 2.7 Quantitative polymerase chain reaction

The isolation of total RNA and complementary DNA (cDNA) synthesis were performed as previously described.^31^ The primer sequences were GAPDH, 5′‐GAAGGTGAAGGTCGGAGTCA‐3′ and 5′‐AATGAAGGGGTCATTGATGG‐3; TF, 5′‐GTCTTCGCCCAGGTGGC‐3′ and 5′‐TGACTTAGTGCTTATTTGAACA GTG‐3′.

### 2.8 Statistical analysis

Data were presented as the mean ± standard deviation (*SD*) and statistical analyses were performed accordingly using the Student's *t* test, paired *t* tests, analysis of variance (ANOVA) and the Mann–Whitney *U* test. All the analyses were performed using Prism 8.0 (GraphPad) and a *p*‐value < .05 was considered to be statistically significant.

## 3 RESULTS

### 3.1 Markers of NETs in the plasma of patients with carotid stenosis

**Figure 1:**
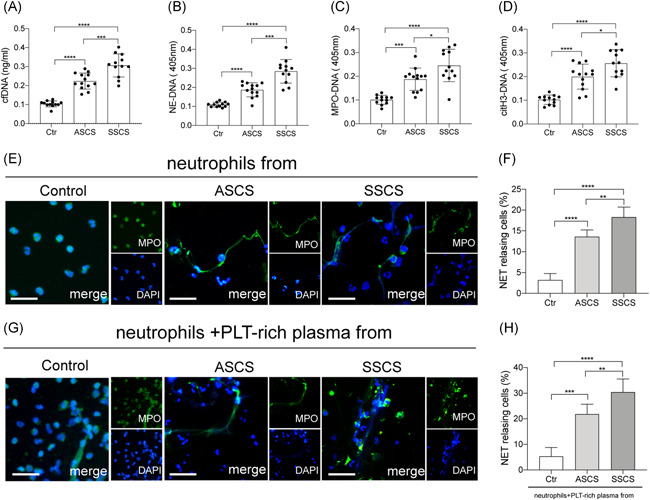
The plasma levels of NET markers increased in symptomatic patients. The NET markers, (A) cfDNA, (B) NE‐DNA, (C) MPO‐DNA, and (D) citH3‐DNA were measured in plasma samples from healthy subjects (*n* = 12), symptomatic patients (*n* = 13), and asymptomatic patients (*n* = 12). (E) Neutrophils from healthy subjects, symptomatic and asymptomatic patients were stained using MPO (green) and DAPI (blue) then analyzed through confocal microscopy. (F) The rate of NETosing neutrophils from each group of patients. (G) Control neutrophils were incubated from PLT‐rich plasma from healthy subjects, symptomatic and asymptomatic patients were stained using MPO (green) and DAPI (blue) then analyzed through confocal microscopy. (H) The rate of NETosing neutrophils from each group of patients. The inset scale bar in E and G are 20 μm. Data are presented as the mean ± SD. cfDNA, cell‐free DNA; citH3‐DNA, citrullinated histone H3‐DNA; DAPI, 4',6‐diamidino‐2‐phenylindole; MPO‐DNA, myeloperoxidase‐DNA; NE‐DNA, neutrophil elastase‐DNA; NET, neutrophil extracellular trap; PLT, platlets. *p < .05, **p < .01, ***p < .001 and ****p < .0001

To investigate the presence of NETs during the progression of carotid stenosis, the study assessed the following markers: Cf‐DNA, myeloperoxidase‐DNA (MPO‐DNA), neutrophil elastase‐DNA (NE‐DNA), and H3cit‐DNA, in the plasma of healthy subjects and asymptomatic as well as symptomatic patients suffering from carotid stenosis, using ELISA (Figure 1A−D). The results revealed a marked increase in the plasma levels of NETs markers in patients with symptomatic carotid stenosis compared with the healthy subjects and asymptomatic patients. In addition, the neutrophils isolated from each group were stained using MPO and citH3 then analyzed through confocal microscopy (Figure 1E,F). The findings showed an increase in the percentage of NETs releasing neutrophils in symptomatic patients with carotid stenosis, in a randomly selected field of the immunofluorescence images, compared with the other groups. Control neutrophils were incubated with PLT‐rich plasma from healthy subjects and patients. PLT‐rich plasma from symptomatic patients induced a higher percentage of NETs observed by confocal microscopy (Figure 1G,H).

### 3.2 Neutrophil‐platelet aggregates promote the generation of NETs in patients with carotid stenosis

**Figure 2:**
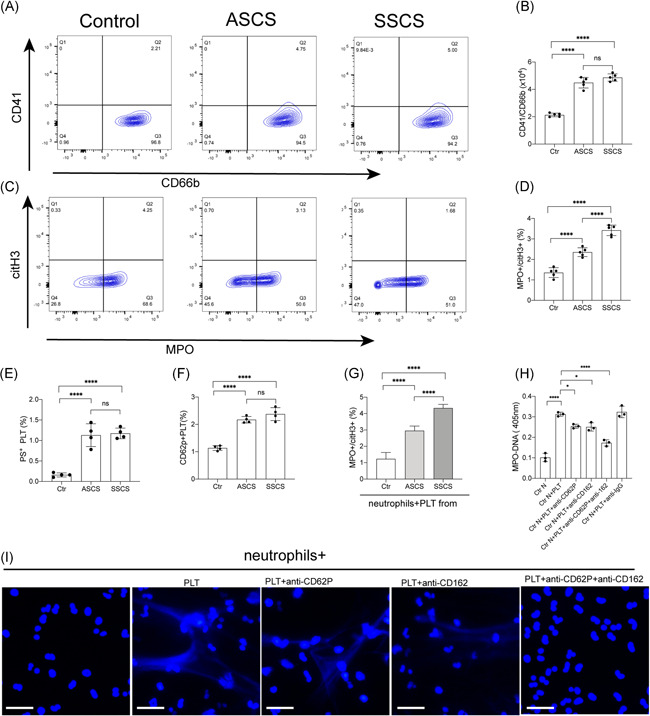
Neutrophil‐platelet (PLT) aggregates enhance the generation of NETs in symptomatic patients. (A) Neutrophil‐platelet aggregates from healthy subjects (*n* = 5), symptomatic patients (*n* = 5), and asymptomatic patients (*n* = 5), defined as CD41/CD66b ^+^ events, were detected using flow cytometry. (B) The rate of neutrophil‐platelet aggregates from each group. (C) NETting neutrophils defined as MPO + /citH3+ events were detected from neutrophils isolated from healthy subjects (*n* = 5), symptomatic patients (*n* = 5), and asymptomatic patients (*n* = 5) then analyzed through flow cytometry. (D) The rate of NETting neutrophils from each group. (E) The rate of PS + platelets and (F) CD62P + platelets from each group. (G) Control neutrophils were treated with control PLTs, platelets from asymptomatic patients and platelets from symptomatic patients. In the inhibition assays, treated neutrophils were incubated with platelets from symptomatic patients, in the presence of anti‐CD62P and CD162 antibodies. MPO‐DNA of patients was detected through ELISA and the generation of NETs (arrow) was stained with DAPI. The inset scale bars in I are 30 μm. Data are presented as the mean ± *SD*. DAPI, 4',6‐diamidino‐2‐phenylindole; ELISA, enzyme‐linked immunosorbent assay; MPO‐DNA, myeloperoxidase‐DNA; NET, neutrophil extracellular traps; PS, phosphatidylserine. *p < .05, **p < .01, ***p < .001 and ****p < .0001

Activation of PLT is a critical aspect in the pathophysiological process of carotid stenosis.^32^, ^33^, ^37^ In addition, platelet adherence to neutrophils was shown to be a threshold switch for the formation of neutrophil extracellular traps.^35^, ^36^, ^38^, ^39^ Therefore, the present study evaluated the potential role of platelets in the generation of NETs in carotid stenosis. The results from flow cytometry showed a significant increase in Neutrophil‐PLT aggregates (defined as CD41^+^/CD66b + events) in patients suffering from carotid stenosis, compared with the healthy subjects (Figure 2A). Moreover, the rate of formation of neutrophil‐PLT aggregates in samples from symptomatic patients was higher than that from asymptomatic patients (Figure 2B). The study also detected the NETting neutrophils, defined as MPO + /citH3+ events, in the same samples. The results showed that the proportion of NETting neutrophils from symptomatic patients was higher than that from asymptomatic patients and healthy subjects (Figure 2C,D). Flow cytometry also showed that symptomatic patients had a higher rate of expression of PS as well as CD62P (P‐selectin) on platelets, among the three groups (Figure 2E,F). Platelets from symptomatic patients showed a highest ability of NETs generation than other groups (Figure 2G). Moreover, control neutrophils were incubated with platelets from symptomatic patients, in the presence of anti‐CD62P and the CD162 (PSGL‐1) antibodies which could inhibit the interaction between neutrophils and platelets. The results showed that these inhibitors could significantly inhibit the generation of NETs, indicating the crucial role of platelets in NETosis, in carotid stenosis (Figure 2H,I).

### 3.3 NETs play a role in the coagulation state of patients with carotid stenosis

**Figure 3:**
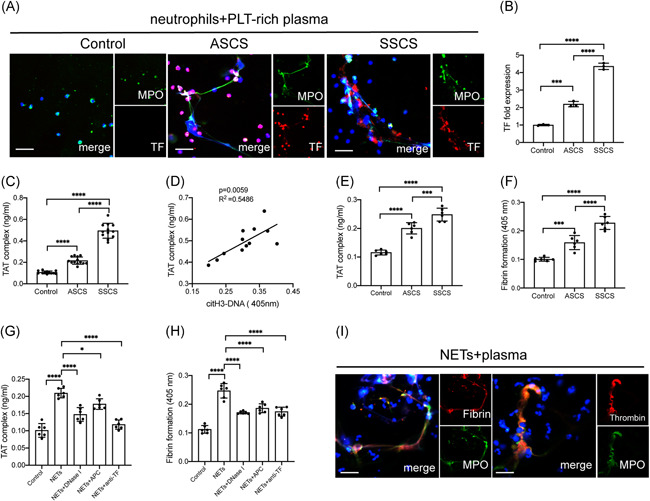
NETs contribute to procoagulant activity by binding with coagulation factors through TF. (A) Control neutrophils were incubated with plasma from healthy subjects and patients with carotid stenosis. Neutrophils treated with ICH plasma expelled extracellular traps (arrowhead) stained with TF (red) and MPO (green). (B) Fold expression of TF mRNA in neutrophils incubated with plasma from controls and patients. (C) Detection of the TAT‐complex in plasma from healthy subjects and patients. (D) The TAT‐complex was positively correlated with H3cit‐DNA in plasma from symptomatic patients. Control plasma were incubated with NETs from controls and patients and the TAT‐complex (E) and fibrin formation (F) were detected. Control plasma was incubated with NETs (0.5 μgDNA/ml) then treated with DNase I, APC, and anti‐TF antibody in the inhibition assays. These inhibitors markedly decreased the thrombin (G) and fibrin formation (H). Colocalization of NETs and thrombin and fibrin were stained with MPO (green) and thrombin (red) and fibrin(red). The inset scale bar in A is 30 μm and I is 2030 μm. The results are expressed as the mean ± *SD*. APC, activated protein C; ICH, intracranial hemorrhage; NET, neutrophil extracellular traps; TF, tissue factor. *p < .05, **p < .01, ***p < .001 and ****p < .0001

It is widely known that carotid artery stenosis is one of the leading causes of stroke.^5^ Additionally, various studies identified NETs as novel therapeutic targets in thrombotic diseases.^40^, ^41^ Consequently, the study examined whether NETs played a role in the coagulation state in carotid stenosis. Therefore, control neutrophils were incubated with plasma from healthy subjects and patients with carotid stenosis. Confocal images showed that neutrophils treated with plasma from symptomatic patients expressed higher levels of the tissue factor than those from the other groups (Figure 3A,B). Interestingly, the high levels of the tissue factor showed colocalization with extracellular traps, indicating the potential procoagulant activity of NETs in symptomatic patients. In addition, levels of the thrombin anti thrombin complex (TAT) complex in plasma from symptomatic patients were higher than those from healthy and asymptomatic patients (Figure 3C). Moreover, the TAT complex was positively correlated with citrullinated histone H3‐DNA (citH3‐DNA) in samples from patients with carotid stenosis especially the symptomatic ones (Figure 3D). Thereafter, control plasma was incubated with NETs from each group. The generation of TAT‐complex and fibrin formation were significantly in samples from symptomatic patients than those from other groups (Figure 3E,F). In the inhibition assays, control plasma was incubated with isolated NETs in the presence of DNase I, APC, and anti‐TF antibody. The results showed a significant decrease in thrombin and fibrin generation when plasma was preincubated with these inhibitors (Figure 3G−I).

### 3.4 NETs induce endothelial dysfunction in carotid stenosis patients

**Figure 4:**
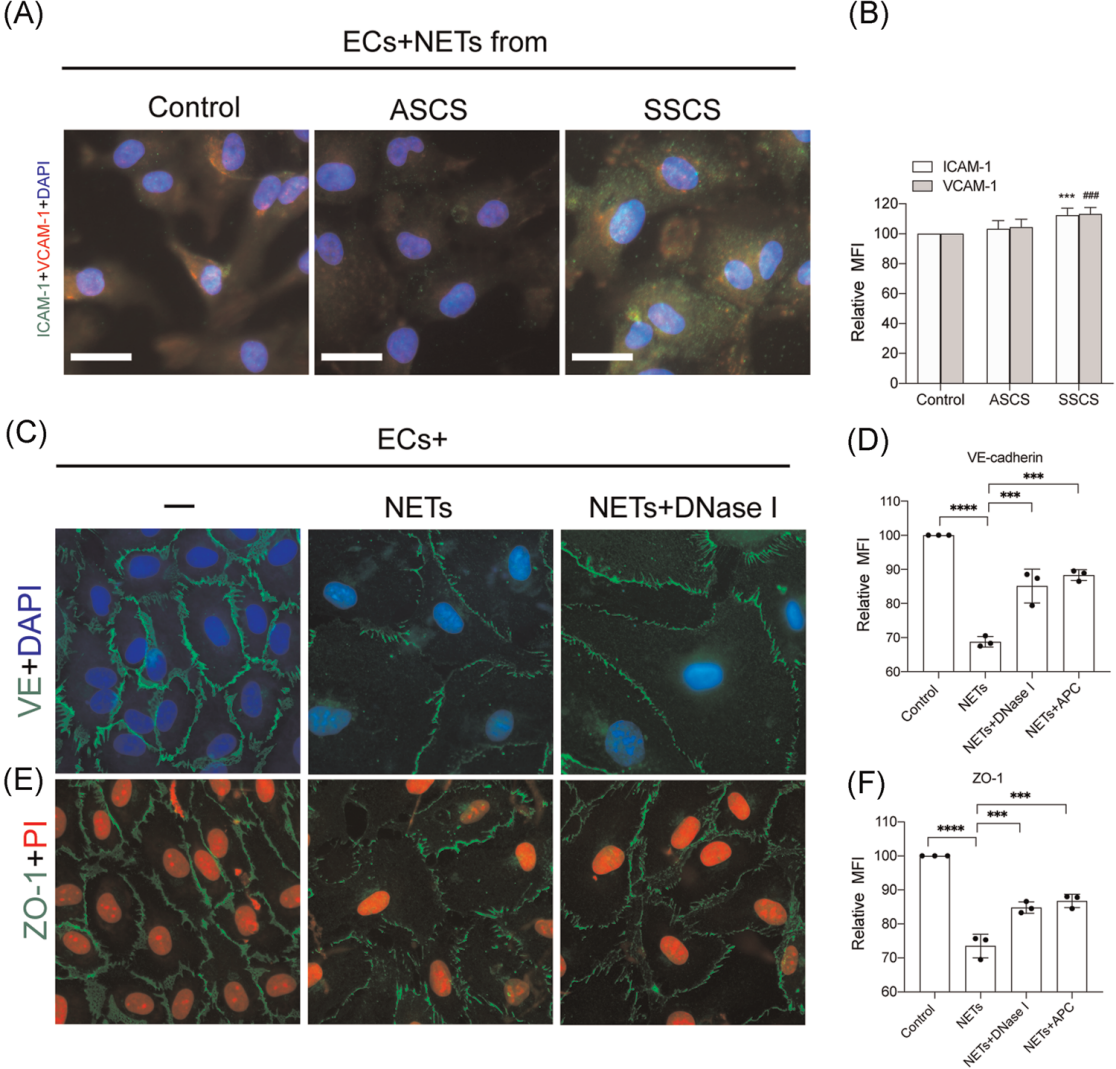
NETs destroyed the endothelial barrier in symptomatic patients. ECs were incubated with control neutrophils and NETs from symptomatic and asymptomatic patients. (A) The expression of ICAM‐1 and VCAM‐1 from treated ECs in each group was analyzed through confocal microscopy. (B) The expression of ICAM‐1 and VCAM‐1 were quantified by MFI. The expression of VE‐cadherin (C) and ZO‐1 (E) in ECs treated with isolated NETs was analyzed through confocal microscopy. There was a decrease in the expression of VE‐cadherin and ZO‐1 when incubated with NETs (0.5 μgDNA/ml) and this cytotoxicity could be reversed by the NETs inhibitors, DNase I, and APC. The expression of VE‐cadherin (D) and ZO‐1 (F) were quantified by MFI. The inset scale bar in A, C, and E are 20 μm. The results are expressed as the mean ± *SD*. APC, activated protein C; EC, endothelial cell; ICAM‐1, intercellular adhesion molecule 1; MFI, mean fluorescence intensity; NET, neutrophil extracellular traps; VCAM‐1, vascular cell adhesion molecule‐1. *p < .05, **p < .01, ***p < .001 and ****p < .0001

**Figure 5:**
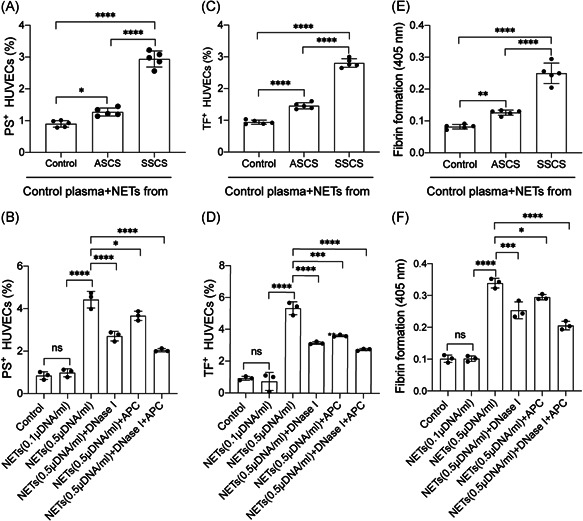
NETs enhanced the procoagulant activity of endothelial cells. ECs were incubated with isolated NETs from controls and healty subjects. There was an increase in the expression of (A) PS and (C) TF in ECs when incubated with NETs from symptomatic patients. In inhibition assays, the expression of PS (C) and TF (D) were decreased by NETs inhibators, DNase I and APC (D) Formation of fibrin in ECs showed a marked elevation when incubated with NETs from symptomatic patients (E) this effect could be reversed by DNase I and APC. The results are expressed as the mean ± *SD*. APC, activated protein C; EC, endothelial cell; NET, neutrophil extracellular traps; PS, phosphatidylserine; TF, tissue factor. *p < .05, **p < .01, ***p < .001 and ****p < .0001

Previous studies strongly associated endothelial activation with the pathophysiology of carotid stenosis.^42^, ^43^ Consequently, the present study investigated the interaction between NETs and ECs in carotid stenosis. Herein, ECs were incubated with NETs from symptomatic and asymptomatic patients. The results revealed a marked elevation in the expression of intercellular adhesion molecule 1 (ICAM‐1) and vascular cell adhesion molecule‐1 (VCAM‐1) in ECs treated with NETs from symptomatic patients (Figure 4A,B). Next, ECs were treated with isolated NETs at different concentrations. The study then examined the expression of VE‐cadherin and ZO‐1 in ECs treated with isolated NETs. The results showed a decrease in the expression of VE‐cadherin and ZO‐1 and this cytotoxicity could be reversed by the NETs inhibitors, DNase I and APC (Figure 4C–F). Furthermore, thrombogenicity of ECs was assessed following the treatment with NETs from healthy subjects and patients. The findings revealed an increase in the expression of PS and TF in ECs with NETs from symptomatic patients and NETs inhibitors, DNase I and APC could decrease the expression of PS and TF on ECs (Figure 5A−D). The results also showed a similar trend in fibrin formation (Figure 5E). Additionally, the formation of fibrin in ECs was significantly decreased in the presence of NETs inhibitors such as DNase I and APC (Figure 5F).

## 4 DISCUSSION

The present study made four major observations. First, the plasma levels of NETs markers were significantly higher in samples from patients with symptomatic carotid stenosis compared with the asymptomatic patients and healthy subjects. Second, platelets played a critical role in the generation of NETs in carotid stenosis. Third, NETs contributed to procoagulant activity by binding with coagulation factors through TF. Fourth, NETs destroyed the endothelial barrier and converted ECs into PCA.

Atherosclerosis is a well‐recognized chronic inflammatory process and macrophages were identified as the major leukocytes in atherosclerotic lesions and therefore considered the key drivers of the development of the disease.^11^ In addition, numerous studies have provided important clues on the possible role of neutrophils in the pathogenesis of Atherosclerosis.^12^, ^13^, ^14^, ^15^, ^16^ Notably, neutrophil counts were proven to be tightly correlated with the presence and severity of coronary and carotid atherosclerotic lesions.^12^, ^13^, ^14^, ^15^, ^16^ Additionally, some studies reported that neutrophils mainly existed in the form of NETs in atherosclerotic lesions.^17^, ^18^, ^19^ NETs are web‐like structures released from activated neutrophils and are composed of histones and neutrophil granule proteins, such as elastase, myeloperoxidase, and cathepsin G.^20^, ^21^, ^22^ Previous studies showed that NETs work together with macrophages to contribute to the development of atherosclerosis.^44^ Therefore, the present study assessed the levels of the markers of NETs. cf‐DNA, MPO‐DNA, NE‐DNA and citH3‐DNA in plasma from healthy subjects, symptomatic and asymptomatic patients suffering from carotid stenosis. The results showed that the markers were present in significantly elevated levels in plasma from patients with symptomatic carotid stenosis, compared with the other groups. Moreover, analysis using confocal microscopy showed that neutrophils from symptomatic patients had the highest ability to generate NETs. Previous studies showed significant differences in the risk of stroke recurrence between asymptomatic and symptomatic patients with severe carotid stenosis.^6^, ^10^ However, the precise mechanisms underlying these differences are yet to be fully elucidated. Furthermore, platelet activation has been well identified in carotid stenosis by numerous studies and plays an important role in the recurrence of stroke.^32^, ^33^, ^37^ In addition, recent studies highlighted the potential role of the neutrophil‐platelet complex in thrombogenicity in symptomatic carotid artery stenosis.^32^, ^33^ In this study, the neutrophil‐platelet complex was examined by detecting the CD41 ^+^ /CD66b cells in samples from each group. The samples from symptomatic patients showed the highest level of the neutrophil‐platelet complex in all groups. Moreover, there was a similarity in NETting cells from the same samples, indicating the potential interaction between platelet activation and formation of NETs. Notably, platelets were identified to play a critical role in the formation of NETs in some diseases.^35^, ^36^, ^38^, ^39^, ^45^ Therefore, the present study investigated the interaction between NETs and platelets. The results showed that neutrophils treated with plasma from symptomatic patients had a higher ability to generate NETs. Additionally, blocking the interaction between platelets and neutrophils using anti‐CD62P and CD162 antibodies, markedly decreased the formation of NETs. This suggested that platelets were important mediators in the generation of NETs in symptomatic patients.

In addition, NETs were reported to play an important role in the pathophysiology of ischemic stroke and identified as important components of the thrombi in ischemic stroke.^24^, ^25^, ^26^ Numerous clinical studies also showed that the extracranial internal carotid artery atherosclerotic occlusive disease is a leading cause of stroke accounting for about 7%–18% of all the stroke incidences.^3^, ^4^, ^5^, ^6^, ^7^, ^8^, ^9^, ^10^ Moreover, the findings from this study suggested that the significant difference in NETs in plasma from symptomatic and asymptomatic patients may be correlated with the differences rates leading to the incidence of stroke. Previous studies showed increased generation of thrombin in samples from patients with symptomatic disease compared with their asymptomatic counterparts, indicating potential thrombogenicity in symptomatic patients.^37^ Similar results were obtained in the present study by detecting the TAT complex in each group. Additionally, the TAT complex was positively correlated with the markers of NETs, suggesting the potential procoagulant activity of NETs. Confocal images also showed that neutrophils incubated with plasma from symptomatic patients expressed higher levels of the tissue factor than those from the other groups. Notably, tissue factor decorated on NETs was reported to initiate coagulation cascades. Therefore, to evaluate the thrombogenicity of NETs in carotid stenosis, control plasma was incubated with neutrophils and isolated NETs. The confocal images revealed a higher percentage of coagulation factors bound to extracellular traps when control plasma was incubated with isolated NETs. Furthermore, inhibition assays were conducted by incubating the control plasma with isolated NETs in the presence of DNase I, APC, and anti‐TF antibody, to inhibit NETs and TF. The results showed a significant decrease in the levels of coagulation factors binding to NETs, when plasma was preincubated with these inhibitors. A similar trend was also observed while detecting the levels of the TAT complex and fibrin formation in plasma incubated with inhibitors.

Moreover, endothelial activation is considered a crucial process in carotid stenosis. According to a previous study, levels of the endothelial biomarker, vwf (von Willebrand factor), were more elevated in the plasma of patients with symptomatic carotid stenosis than in the asymptomatic ones.^42^, ^43^ The results herein also showed that abnormal generation of NETs could destroy the endothelial barrier by dysregulating the expression of VE‐cadherin and ZO‐1. In addition, the findings revealed the presence of EC thrombogenicity after treatment with different concentrations of NETs. Additionally, treated endothelial cells upregulated PS and TF, which could offer coagulation binding sites for clotting factors, hence initiating coagulation cascades. Furthermore, NETs were shown to induce procoagulant activity in endothelial cells, aggravating thrombosis in some cases.^26^, ^29^ The present study also showed that the procoagulant activity could be inhibited by DNase I and APC which are NETs inhibitors.

In summary, the study demonstrated that platelet activation induced the generation of NETs in patients with carotid stenosis. Additionally, analysis of the interactions between NETs and ECs may help explain differences in the incidence of stroke between symptomatic and asymptomatic patients. Therefore, NETs may provide new potential targets for the prevention and treatment of vascular complications in patients with severe carotid stenosis.

## CONFLICT OF INTERESTS

The authors declare that there are no conflict of interests.

## AUTHORS CONTRIBUTIONS

Shihua Zhang, Mengfan Guo, and Yankun Cui designed the study, analyzed the data, made the figures and wrote the paper. Qianzi Liu and Jingfeng Liu conducted the assays and provide the study materials or patients. All authors approval of final manuscript.
